# Morphological plasticity of ectomycorrhizal short roots in *Betula* sp and *Picea abies* forests across climate and forest succession gradients: its role in changing environments

**DOI:** 10.3389/fpls.2013.00335

**Published:** 2013-09-02

**Authors:** Ivika Ostonen, Katrin Rosenvald, Heljä-Sisko Helmisaari, Douglas Godbold, Kaarin Parts, Veiko Uri, Krista Lõhmus

**Affiliations:** ^1^Institute of Ecology and Earth Sciences, University of TartuTartu, Estonia; ^2^Department of Forest Sciences, University of HelsinkiHelsinki, Finland; ^3^BOKU, Institute of Forest EcologyVienna, Austria; ^4^Institute of Forestry and Rural Engineering, Estonian University of Life SciencesTartu, Estonia

**Keywords:** plasticity, ectomycorrhizal roots, morphological characteristics, root acclimation, silver birch, Norway spruce, forest succession, climate gradient

## Abstract

Morphological plasticity of ectomycorrhizal (EcM) short roots (known also as first and second order roots with primary development) allows trees to adjust their water and nutrient uptake to local environmental conditions. The morphological traits (MTs) of short-living EcM roots, such as specific root length (SRL) and area, root tip frequency per mass unit (RTF), root tissue density, as well as mean diameter, length, and mass of the root tips, are good indicators of acclimation. We investigated the role of EcM root morphological plasticity across the climate gradient (48–68°N) in Norway spruce (*Picea abies* (L.) Karst) and (53–66°N) birch (*Betula pendula* Roth.,* B. pubescens* Ehrh.) forests, as well as in primary and secondary successional birch forests assuming higher plasticity of a respective root trait to reflect higher relevance of that characteristic in acclimation process. We hypothesized that although the morphological plasticity of EcM roots is subject to the abiotic and biotic environmental conditions in the changing climate; the tools to achieve the appropriate morphological acclimation are tree species-specific. Long-term (1994–2010) measurements of EcM roots morphology strongly imply that tree species have different acclimation-indicative root traits in response to changing environments. Birch EcM roots acclimated along latitude by changing mostly SRL [plasticity index (PI) = 0.60], while spruce EcM roots became adjusted by modifying RTF (PI = 0.68). Silver birch as a pioneer species must have a broader tolerance to environmental conditions across various environments; however, the mean PI of all MTs did not differ between early-successional birch and late-successional spruce. The differences between species in SRL, and RTF, diameter, and length decreased southward, toward temperate forests with more favorable growth conditions. EcM root traits reflected root-rhizosphere succession across forest succession stages.

## Introduction

The development of an efficient root system is necessary for trees to ensure sufficient nutrient uptake in various conditions. Hence, trees must acclimate by modifying either the biomass of fine roots or the morphology and physiological activity of nutrient absorbing root tips or both (Lõhmus et al., 2006). Morphological plasticity of roots with primary development (generally first (youngest) and second branching order roots) is the fastest mechanism for root acclimation in trees. A clear morphological response of ectomycorrhizal root tips has been shown along latitudinal gradient of Norway spruce forests (Ostonen et al., 2011) as well as in long-term soil temperature and nutrient manipulation experiments (Leppälammi-Kujansuu et al., 2013). Morphological plasticity of roots within species can be defined as the response range of root traits to different environments. The traits of roots with primary structure are important for optimizing the mineral nutrition of the plant (Curt and Prévosto, 2003; Comas and Eissenstat, 2004) although they may vary considerably within a family, a genus, and even within a species (Ostonen et al., 2007a,b; Francini and Sebastiani, 2010), and also with tree age (Rosenvald et al., 2011a, 2013).

Root trait values of different genotypes within a tree species in changing environment can be considered as reaction norms in wider sense. Plasticity response is higher the further away the stand is from the optimum growth condition (Ghalambor et al., 2007). Since the structure of EcM roots is closely related to root functions, different morphological and functional root parameters are potential indicators of the nutrition of trees in relation to soil conditions. Most commonly used root traits to characterize the morphological response to the local environment are specific root area (SRA, m^2^ kg^−1^), specific root length (SRL, m g^−1^) and root tissue density (RTD, kg m^−3^), but also mean diameter and length of root tips (used here as synonym to EcM roots) (Fitter, 1985; Lõhmus et al., 1989, Ostonen et al., 1999; Comas and Eissenstat, 2004; Guo et al., 2004; Leuschner et al., 2004; Ostonen et al., 2006, 2007a,b).

High SRL and SRA values are believed to be the morphological tool of roots to increase the surface area, which may lead to improvement in nutrient acquisition (Schippers and Olff, 2000). Furthermore, morphological plasticity of the primary root tips enables trees to survive in primary succession stands, for example following oil-shale mining (Ostonen et al., 2006; Kuznetsova et al., 2010; Rosenvald et al., 2011a), where the pedogenesis only started on detritus (Reintam, 2004). Trees show high SRL and SRA of root tips at young age (Lõhmus et al., 2006; Rosenvald et al., 2011a, 2013), whereby the changes in EcM root morphology of silver birch occur faster at younger age-before the age of 10 years (Rosenvald et al., 2011a, 2013). However, the examples of forests acclimation via plasticity of EcM root tips and general trends in the fine root system over time, e.g., in a chronosequence or in different successional stages of forest are very seldom documented.

*Picea abies* is one of the dominant conifer species in the late stage of forest succession in boreal forests, while the deciduous *Betula* species tend to occur in the early stages of forest succession in Northern Europe and Russia. The environmental gradient from boreal to temperate spruce and birch forests includes substantial changes in growing conditions—increasing mean annual temperature, length of growing season etc., as well as rise in anthropogenic N deposition, and land use intensity. Climate change induced climatic alterations similar to those taking place along latitudinal gradient within the territory ranging from northern boreal to temperate forests are expected to occur in time in northern Europe.

The birch forests (predominantly *Betula pendula* and some mixed stands of *B. pendula* and *B. pubescens*) investigated in our study, include primary and secondary successional stands growing on reclaimed mining areas, afforested abandoned fields and in forest areas. The spruce forests, on the other hand, range from poor to the most fertile sites replacing the broadleaved trees from mid-successional stages in the boreal and temperate zone.

We show morphological plasticity of ectomycorrhizal root tips in Norway spruce and birch to be an active mechanism in root acclimation to site conditions; larger plasticity may enhance root acclimation capacity to local environmental conditions in short-term and adaptation capacity to climatic changes in long-term scale. We considered EcM morphological plasticity as the response of certain morphological parameters to different site conditions and stand age. To examine the greatest possible extent of the morphological plasticity, stands of very different growth conditions were included in our meta-study.

We hypothesized that:

1. Late-successional and pioneer tree species have different root tip morphological plasticity and strategies to cope with colder climate with shorter vegetation period, and show distinctive acclimation patterns along the latitudinal gradient and forest zones.
2. High level of environmental stress, as exists in northern boreal forests and initially in primary successional birch forests, leads to extensive alteration in EcM root morphology.

The specific aims were to find out the tree species-specific EcM root traits being most indicative and responsive in morphological acclimation across environmental gradients concurrent in forest succession.

## Materials and methods

In order to analyse the morphological plasticity of EcM roots of an early-successional and a late-successional tree species, we compiled a database of morphological characteristics measured for EcM roots originating from 14 Norway spruce (*Picea abies*) and 30 birch (*Betula pendula* and *B. pubescens*) stands (all silver birch stands except two mixed stands in Finland: Olkilouto and Punkaharju), partly published earlier (Supplementary Tables 1, 2). Data of control plots were used in case of manipulation experiments (Leppälammi-Kujansuu et al., 2013; Parts et al., in preparation).

**Figure 1:**
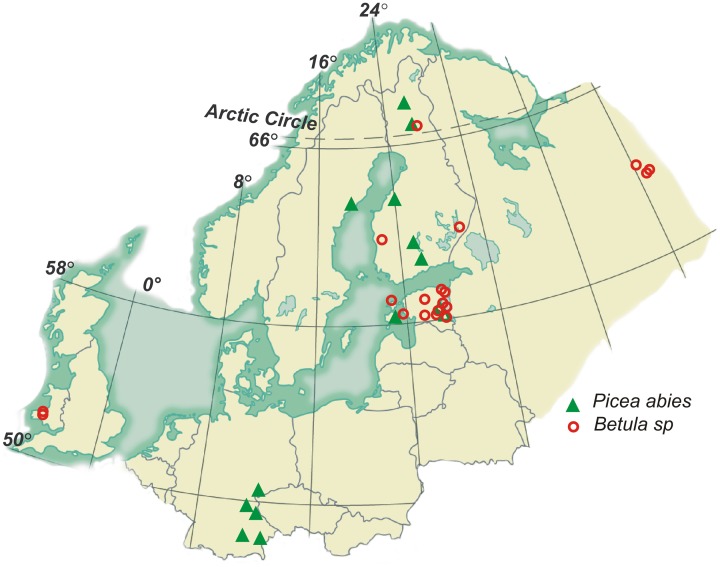
**Locations of the study areas**.

The spruce stands covered a latitudinal range from 48 to 68°N including 5 temperate, 3 hemi-boreal, and 6 boreal stands in age from 30 to 140 years. The birch stands covered a latitudinal range from 53 to 66°N and a longitudinal range from 2°W to 51°E and included 6 native forest stands in boreal, 8 in boreo-nemoral, and 1 in the temperate zone (Figure 1). Six birch stands of different ages were growing in reclaimed mining areas with stony and alkaline soil, and 9 young birch stands in abandoned agricultural fields. To analyse changes in root morphological traits (MTs) across forest succession gradient, we divided birch stands into three groups: primary successional stands on alkaline quarry detritus exposed by mining (mine forest), secondary successional stands in abandoned agricultural fields (field forest) and in long-term forests on fertile soil (native forest). Age-driven morphological plasticity was analyzed in two silver birch chronosequences growing in mine area and on native forest land in Estonia (included stands are indicated by asterisks in Supplementary table 2). Stands included in native forest chronosequence belong to the *Oxalis* forest site type (Paal, 1997), characterized by high productivity (C/N = 12–23) and acidic soil, and are naturally regenerated; whereas stands growing in a mining area were plantations. To calculate PI-s of root traits for young (<10 years) and all older silver birch, trait means of all 30 birch stands and 46 measurements were used.

In order to be able to compare the plasticity of EcM roots between the tree species, the effect of previous land use was excluded by using only native forest stands, which were older than 10 for birch and 30 years for spruce i.e., have already undergone fast age-induced changes in EcM root morphology (Rosenvald et al., 2013). The EcM root traits were measured in several consecutive years for 11 birch and 7 spruce stands. In the same areas repeatedly measured morphological root traits were used to analyse the effect of study year in both tree species. However, to assess reaction norm of EcM root parameters in silver birch and Norway spruce forests across an environmental gradient, the mean of all years for a site was used.

The study sites for both tree species between latitudes 68 and 48°N display gradients in climate (e.g., mean annual temperature and precipitation, growing season length) as well as in N deposition (for some spruce stands see Ostonen et al., 2011). The different fertility of studied spruce sites was reflected by soil C/N ratio. In general, the southern stands displayed higher fertility. Silver birch stands on native forest land belonged all to the fertile forest site types.

Assuming the increase of RTD and mass (W, mg) of EcM root tips through aging, we analyzed the frequency distributions of RTD and W of root tips in two most northern (Kivalo and Pallasjärvi) and two most southern (Höglwald and Altötting) sites collected in 2008 for spruce (Ostonen et al., 2011). For birch, we compared the frequency distributions of RTD and root tip mass for EcM roots from two northern (Kivalo and two older sites in Syktykvar) and from two southern (Risley Moss, Erastvere) sites, samples were collected in 2009.

### Morphological characteristics and PI calculation

EcM roots were sampled at the end of the growing season (during September and October) by the same methodology throughout different years (Supplementary Table 1). Ten root samples per stand were collected with a spade from the organic layer and up to 20-cm-deep mineral soil layers at random locations. Two or three random EcM root subsamples (20–30 EcM root tips) were taken from each sample to measure the length, diameter, and projection area of root tips using WinRHIZO™ Pro 2003b (Regent Instruments Inc. 2003). The number of short root tips per sample collection of a stand ranged from 234 to 949 for spruce and from 239 to over 1000 for birch. The EcM root tips were washed cleaning with a small soft brush, to remove all soil particles, and counted under a microscope after separation from the long roots. The presence of ectomycorrhizal infection was recorded, >97% of the analyzed root tips were ectomycorrhizal. As the EcM root tips were taken randomly from the subsamples, the mean values of root MTs per stand reflected the site-specific proportion of different morphotypes.

The air-dry root tips were dried at 70°C for 2–3 h to constant weight and weighed to 0.01 mg. Root tissue density (kg m^−3^), SRA, (m^2^ kg^−1^) and SRL (m g^−1^) were calculated as *M*/*V*, *S*/*M*, and *L*/*M*, where *S*,* M*, and *L* are mean root tip surface area, dry mass and length, respectively. Root tip frequency was expressed as the number of root tips per 1 mg of dry mass (RTF, No/mg). The methods for determining root MTs are given in detail by Ostonen et al. (1999).

Plasticity index (PI) was calculated as follows:

$$\text{PI}=\left(X_{\text{max}}-X_{\text{min}}\right)/X_{\text{max}}$$

where *X*_max_—maximum stand mean, *X*_min_—minimum stand mean in the sample of the analyzed stands.

The reaction norms were expressed as the functional relations between values of a root trait and an environmental parameter. Plasticity of mean EcM root MT of the i-th stand in response to particular environmental conditions expressed through latitude (L) was defined as the absolute value of the slope of the reaction norm between MT and L (e.g., Scheiner, 1993; Pigliucci and Schlichting, 1998; Lepik et al., 2005). Such a plasticity estimate is comparable across different traits and species (Lepik et al., 2005).

To diminish age and land type effect species–specific plasticity was evaluated for older (>10 years) stands growing on native forest land.

## Statistics

Repeated measures ANOVA was used on repeatedly measured data of native forest stands to evaluate the influence of measurement year on EcM root morphology. General Linear Models analyses (GLM) were applied to prove the impact of tree age and succession type (mine, field, and native forest) on EcM root tip parameters.

Redundancy analysis (RDA) (CANOCO program; ter Braak and Smilauer, 2002) was used to detect and visualize relationships between the root morphological characteristics (7) and sites (44) and between latitude, C/N, pH and stand age. The significance of RDA results was tested with a permutation test (*P* < 0.01).

*T*-test was applied to control the differences in trait means and PI values between of spruce and birch, and between birch successional types. Simple regression model was used to estimate the relationships between means of SRL and RTF and the location of stands. Forward stepwise regression analysis was used to reveal which parameters explain best the variation of SRL and RTF.

## Results

### Plasticity of EcM roots in silver birch, the influence of forest successional type and stand age

As the collected birch data were obtained from stands with different age, site, and climate conditions, the results show the variation range of EcM root tip morphology of silver birch (Table 1). Plasticity indices were higher for functional EcM root parameters (SRA, SRL and root tissue density) of birch; the lowest plasticity showed EcM root diameter.

**Table 1:** **The means of stand means and variation between stand means of morphological characteristics of EcM roots of silver birch**.

**EcM root parameter**	**Mean**	**Min**	**Max**	**CV**	**PI**
SRL (m/g)	91.2	44.6	313.9	0.61	0.86
SRA (m^2^/kg)	82.5	45.1	274.6	0.50	0.84
RTD (kg/m^3^)	186	54	279	0.31	0.81
Diameter (mm)	0.310	0.212	0.413	0.15	0.49
Length (mm)	1.24	0.76	2.65	0.27	0.71
Mass (mg)	0.0165	0.0077	0.0279	0.31	0.72
RTF (No/mg)	73.4	39.4	143.1	0.32	0.73

All 31 silver birch stands (characteristics are shown in Supplementary table 2) and 46 measurements are included. Abbreviations: CV, coefficient of variation; PI, plasticity index of stand means.

**Figure 2:**
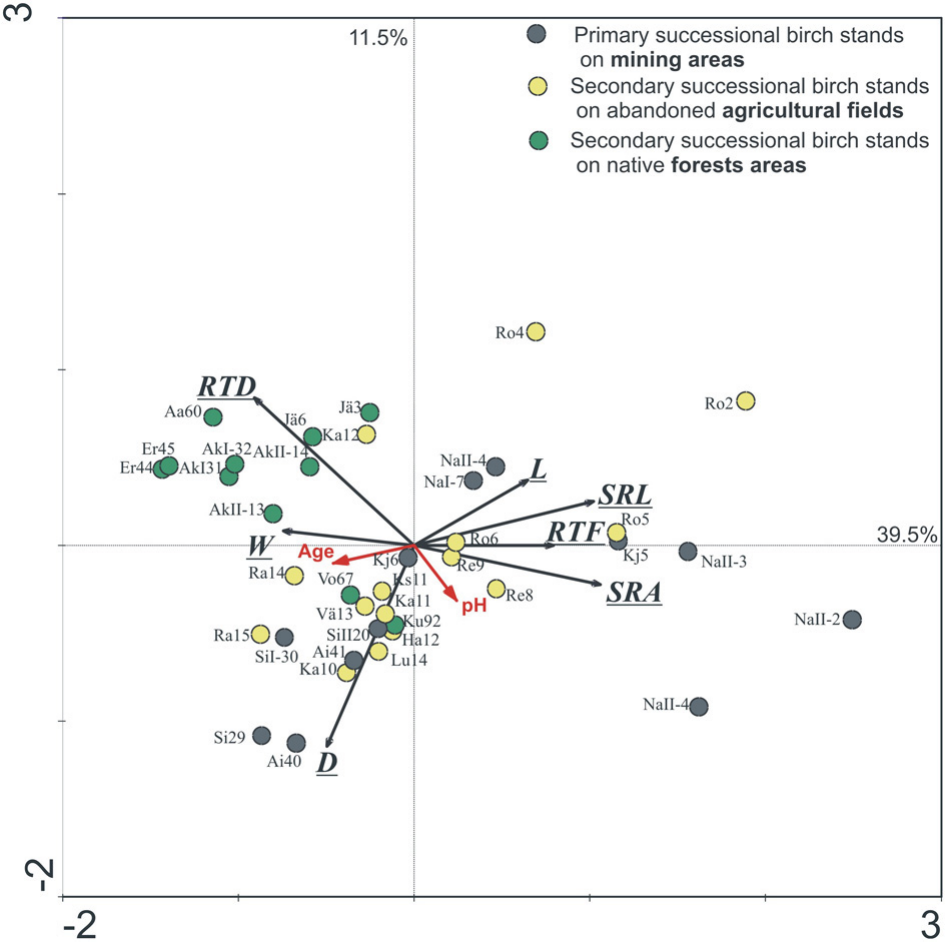
**The Estonian birch stands on the ordination plot based on redundancy analyses of EcM root tip morphological parameters [SRL, SRA, root tissue density (RTD), diameter (D), length (L), mass (M), and root tip frequency per mass unit (RTF, No/mg)].** Numbers after label show the age of the stand. The sites and stand age account for 56.2% of total variability. Axis 1 describes 39.5% and axis 2 describes 11.5% of the total morphological variation, *p* = 0.001. Abbreviations of sites are shown in Supplementary Table 1.

The effect of tree age and stand successional type (mine forest as primary successional forests, field and native forests as secondary successional forests) on EcM root morphology of silver birch was illustrated in an ordination biplot based on RDA of EcM root morphological parameters, where only Estonian stands were included in order to exclude the influence of climate (Figure 2). Tree age and forest successional type both affected significantly EcM root tip traits of silver birch (GLM, *p* < 0.01). Native forest stands formed a separate group also in the RDA ordination plot (Figure 2). Mine site forests had the biggest between-site variation because great changes occurred in soil conditions during stand development on very stony and alkaline mine spoil in primary succession (Rosenvald et al., 2011a). In the ordination plot, stand age increased along the first axis for all three forest succession types. EcM root SRL, SRA, length, and RTF decreased with tree age, whereas mean diameter, mass, and RTD increased with stand age (Figure 2). Changes in root traits across sites in the ordination plot reflect also soil and rhizosphere succession—mine and field stands are likely to develop toward the fertile native forest stands. Plasticity indices of root traits of stands belonging to the fertile silver birch chronosequence of native forest (0.21–0.56, mean PI = 0.37) is lower than that of the mine chronosequence (PI = 0.34–0.85, mean PI = 0.65), where young birch stands have harsh soil conditions (Table 2).

**Table 2:** **Plasticity indices (PI) of root parameters of stands belonging to two silver birch chronosequences: a chronosequence of fertile stands on native forest land belonging to the same forest type (*Oxalis*) (3–60 years old stands) and recultivated mine area stands (1–41 years old)**.

**EcM root parameters**	**Silver birch chronosequences**	
	**PI Native forest**	**PI Mining area**
SRL (m/g)	0.317	0.777
SRA (m^2^/kg)	0.456	0.845
RTD (kg/m^3^)	0.215	0.676
Diameter (mm)	0.206	0.344
Length (mm)	0.295	0.712
Mass (mg)	0.546	0.570
RTF (No/mg)	0.563	0.600
Mean	0.371	0.646

N = 9 in the forest chronosequence, and N = 9 in the mine area chronosequence.

**Figure 3:**
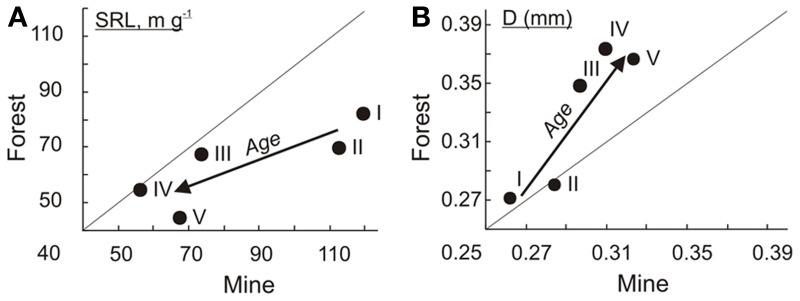
**Mean values of birch EcM root SRL (A) and diameter (B) in five different ages classes (I:3, II:6–7, III:14–20, IV:30–32,V:41–45 years.) in forest vs on mine area.** The arrows indicate the direction of increasing stand age.

In the mine area, 3-years-old (119 ± 7 m/g) and 6-7-years old (113 ± 9) birches have both higher (*t*-test, *p* < 0.01) SRL compared to the same age birches growing in fertile native forest (82 ± 4 and 70 ± 5, respectively). Mean SRL values of EcM roots of silver birch chronosequences in fertile natural forest and in mine area with stony alkaline soil became more similar in older forests (Figure 3A). However, the difference in mean diameter between two chronosequences increases by tree ageing (Figure 3B). Young birches have similar mean D of EcM roots (0.26–0.28 mm) in both successional type, but root D of birches belonging to the three older age groups is thicker in native forest compared to respective age groups of mine area (*t*-test, *p* < 0.01).

Morphological response to different environmental conditions of young (≤10 years) birches was generally higher compared to older (>10years) birch trees (Figure 2, Table 3) except PI of EcM root D. PI-s of functional parameters of young stands were especially high (>0.8). PI values did not vary much between parameters inside the group of older birch stands (0.44–0.60). However, SRL had again the highest PI there.

**Table 3:** **Plasticity indices (PI) of EcM root parameters for the young (*N* = 17) and older (*N* = 29) age groups of silver birch**.

	**Young birches**	**Older birches**
	**(≤10 yrs.) PI**	**(11–92 yrs.) PI**
SRL (m/g)	0.829	0.598
SRA (m^2^/kg)	0.820	0.442
Density (kg/m^3^)	0.806	0.478
Diameter (mm)	0.425	0.474
Length (mm)	0.684	0.483
Mass (mg)	0.684	0.530
RTF (No/mg)	0.660	0.536
Mean	0.699	0.517

### Species-specific plasticity of EcM roots: comparison of silver birch and norway spruce

#### Variation of EcM root traits

The greatest difference between birch and spruce EcM root morphology was in mean mass of root tip, which was more than two times higher for spruce (Table 4). The reason of that is that EcM roots of spruce are longer and thicker, because EcM root tissue density did not differ between species. Birch has higher EcM root SRL and SRA values and lower diameter.

**Table 4:** **The means and variation between stand means of morphological characteristics of EcM root tips and their PI values in silver birch and Norway spruce forests**.

**EcM root tip parameter**	**Species**	**Mean**	**Minimum**	**Maximum**	**Variation coefficient**	**PI**
SRL (m/g)	birch	**65.5**	44.6	111.1	25.2	0.60
	spruce	**42.1**	34.6	57.2	13.5	**0.40**
SRA (m^2^/kg)	birch	**59.8**	45.1	80.8	17.7	0.44
	spruce	**45.3**	37.8	55.3	10.0	0.32
Root tissue density (kg/m^3^)	birch	235	151	279	17.4	0.46
	spruce	261	179	353	17.8	0.49
Diameter (mm)	birch	**0.306**	0.217	0.354	11.4	0.39
	spruce	**0.357**	0.279	0.436	11.2	0.36
Length (mm)	birch	**1.22**	0.99	1.48	12.6	0.33
	spruce	**1.85**	1.10	2.79	24.5	0.60
Mass (mg)	birch	**0.0207**	0.0133	0.0279	21.3	0.52
	spruce	**0.047**	0.0284	0.0766	30.6	0.63
RTF (No/mg)	birch	**56.7**	39.4	80.8	22.0	0.51
	spruce	**25.4**	13.1	41.2	28.7	0.68

Only native forests older than 10 years were included in calculations. N = 16 for silver birch, and N = 23 for Norway spruce; 12 and 14 different forest sites were included respectively. Differences (t-test, p < 0.05) between means of birch and spruce EcM root parameters are indicated in bold.

Mean PI of EcM root traits did not differ (pairwise *t*-test, *p* > 0.05) between birch and spruce, 0.46 and 0.50, respectively. However, root traits with highest PI values differed between tree species: birch had highest PI values for SRL and spruce for root tip frequency (Table 4). PI and also variation of diameter of EcM root tips was small for both species. The biggest difference between species appeared in EcM root length, which had for Norway spruce roughly twice higher PI and variation coefficient than for silver birch. PI indexes and variation coefficients were well-correlated (*r* = 0.96 for birch and *r* = 0.98 for spuce, *p* < 0.01 in both cases) (Tables 3, 4).

#### The influence of forest zone on EcM traits

**Figure 4:**
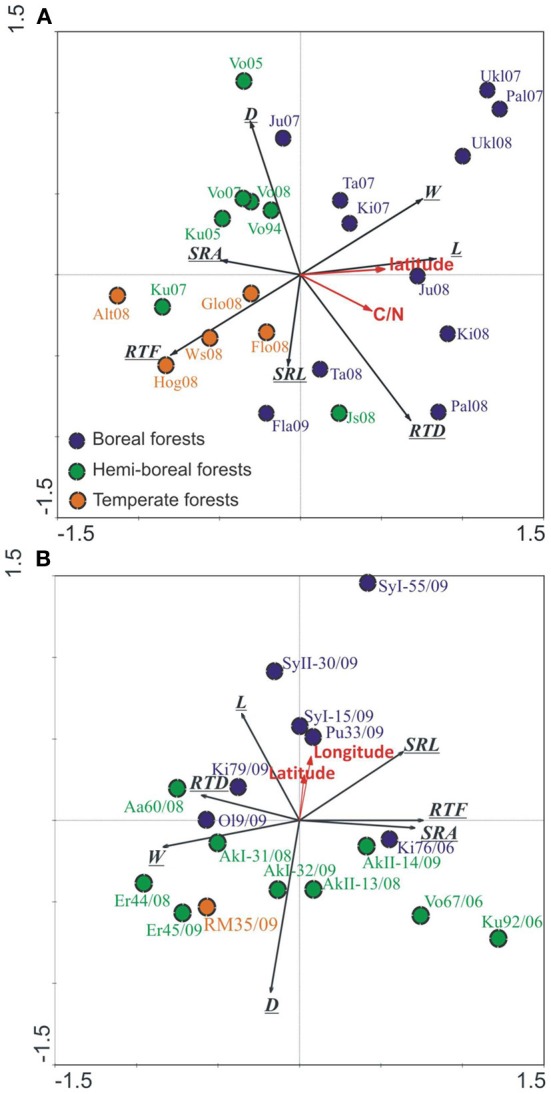
**Ordination of the spruce (A) and birch (B) forests (older than 10 years) on native forest land based on EcM morfological parameters [SRL, SRA, tissue density (RTD), diameter (D), length (L), mass (M), and tip frequency (RTF)].** Norway spruce forests were separated to boreal (blue, >60°N), hemi-boreal (green) and temperate (red) forests. Silver birch stands were separated to boreal (blue, >60°N) and hemiboreal as well as temperate forests (red, <60°N). Latitude, soil C/N ratio and sites accounted for 32.9% of total variability of EcM morphological traits in spruce forests, Axis1 described 18% and Axis2 9.9%, respectively, *p* = 0.001. In birch forests, the latitude, longitude and sites accounted for 23.9% of total variability of EcM morphological traits, axis 1 described 12.4% and axis 2 7.3%, respectively, *p* = 0.001.

The morphology of EcM roots across the climate gradient from subarctic to temperate forests measured in different study years varied greatly for both tree species (Figures 4A,B). Based on the RDA, the sites, geographical location explained 33 and 24% of the variation in EcM root tips morphology in spruce and birch, respectively. The northern boreal forests grouped separately from hemi-boreal and temperate sites on the ordination biplot. Spruce sites located along the first axis, which correlated best with the root tip length, SRA and RTF, and explained about 18% of the total morphological variation (Figure 4A). SRL and D of EcM roots of spruce correlated best with the second axis, which seems to be related to the study year—the stands studied in 2008 locate below the first axis toward the decrease in D, the stands studied in 2007 located above the first axis (Figure 4A). Birch forests located along second axis, which correlated with D and again with the length of root tips (Figure 4B). SRA, RTD, root tip mass and RTF correlated best with the first axis explaining 12% of the total variation in EcM root morphology.

In both tree species, EcM root tips are longer in northern sites, and in the case of birch also thinner. If in the spruce forests, we have clear gradient in edaphic conditions, from low fertility soils in northern stands to high fertility soils in southern stands—the C/N ratio in soil decreased more than two times (Supplementary Table 1) and correlated with latitude (*r* = 0.67, *p* < 0.01), then birch forests were all of high fertility.

**Figure 5:**
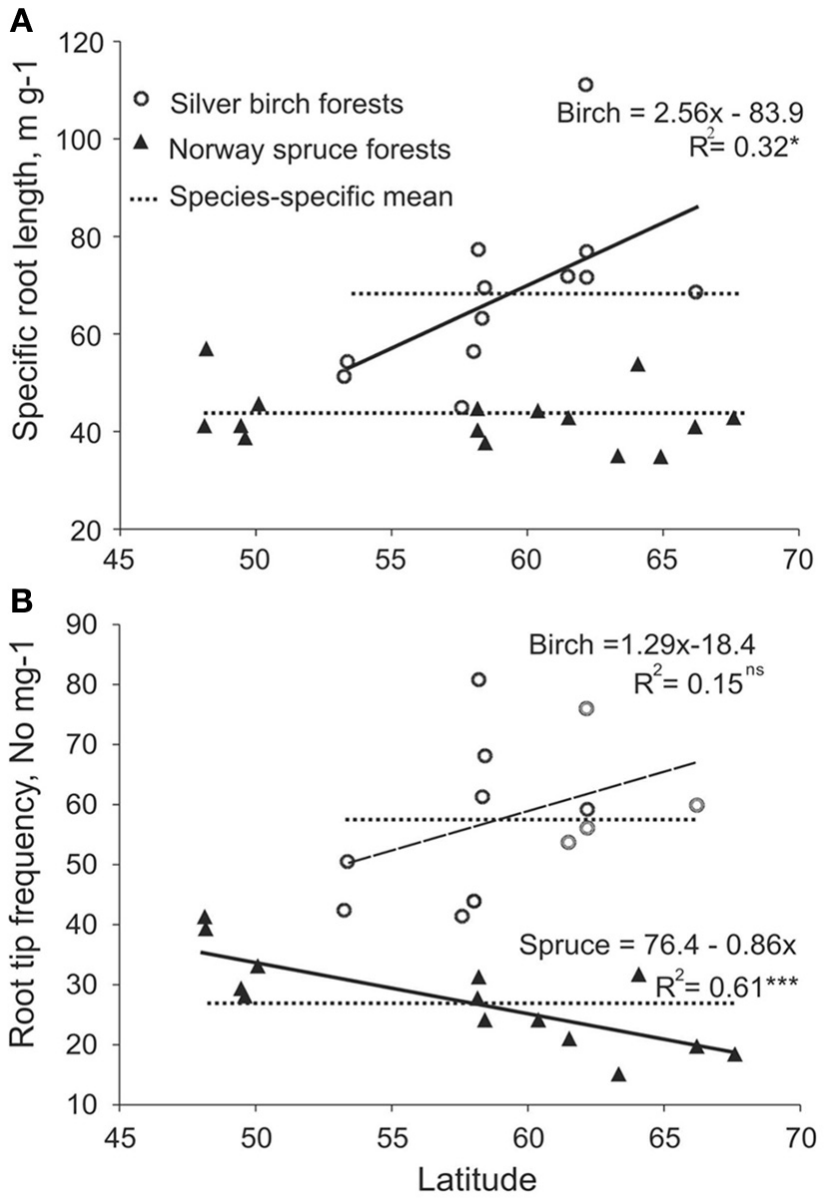
**Reaction norms of (A) specific root length and (B) root tip frequency per mg EcM roots of spruce (filled triangles) and birch (open circles) across the latitudinal gradient**.

We estimated the plasticity using reaction norms across forest zones for SRL and root tip frequency as most highly responsive traits on the basis of root traits PI values (Table 4) and RDA analysis (Figures 4A,B). When subjected to harsher conditions in higher latitudes, the birch increases the SRL of EcM roots, while spruce EcM roots showed no plasticity for this particular trait (Figure 5A). Although RTF expressed considerable plasticity across the latitudinal gradient in both spruce and birch, the tree species had reversed slopes of the regression line (i.e., a different response to environment). The spruce EcM roots showed decreasing and the birch EcM roots increasing RTF toward the northern forests (Figures 5A,B). Though in the case of birch, the regression was not significant due to shorter latitudinal gradient.

**Figure 6:**
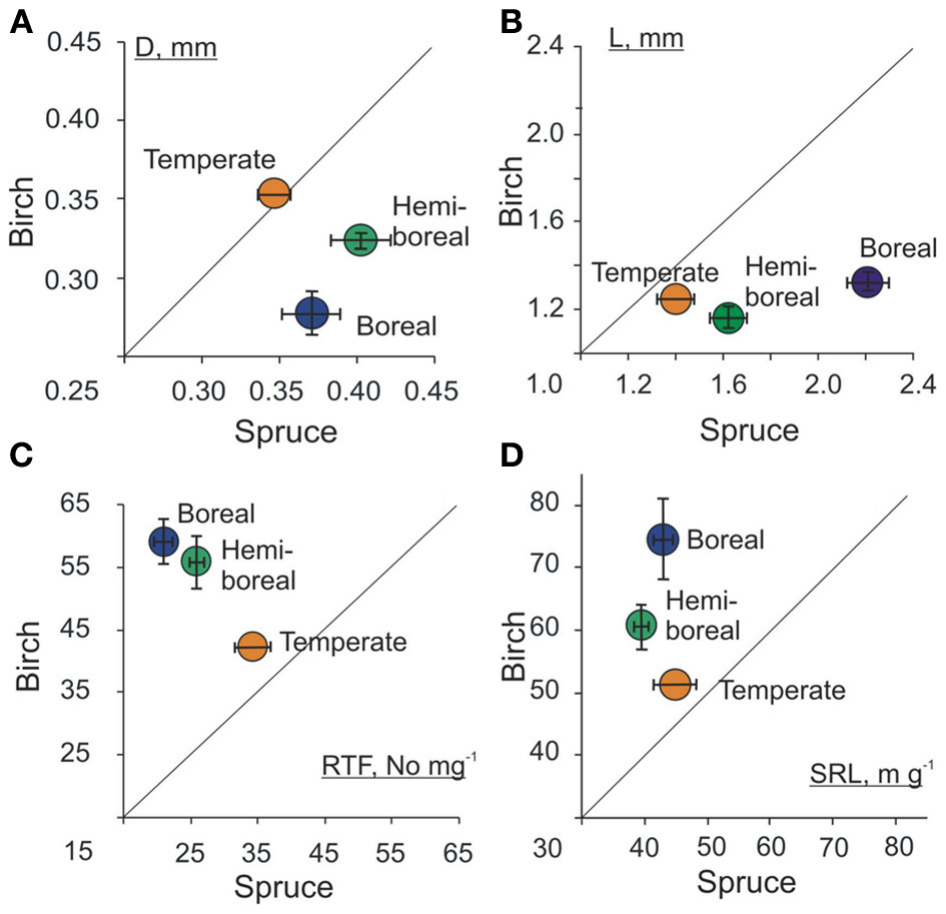
**Mean values of morphological traits (A) diameter (D, mm), (B) length (L, mm), (C) root tip frequency per mass unit (RTF No mg^−1^) and (D) specific root length (SRL, m g^−1^) of birch and spruce in three forest zones: boreal, hemi-boreal, and temperate.** Shown are average values and standard errors of the sample. For birch forests, only older than 10 years stands growing on native forest land were included and only one stand from temperate zone was included.

When the mean values of a root trait of birch are plotted against the values of spruce in boreal, hemi-boreal, and temperate native forests (Figures 6A–D), a remarkable trend appears—the morphology of the studied tree species becomes more similar toward southern forests. Although the values of EcM root tip SRL, RTF, D and L differed between species more in boreal forests, the mean RTD of root tips did not show clear trend in terms of forest zones.

The functional traits SRL and RTF both depend on root D, L and RTD. Forward stepwise regression analysis showed that the root tip frequency was mostly explained by root tip length for spruce (*r*^2^ = 0.80, *p* < 0.001) and SRL by variation of D for birch (*r*^2^ = 0.61, *p* < 0.001).

The study year had significant effect on RTD and SRA of spruce EcM roots (Repeated measures ANOVA; *n* = 6 boreal spruce forests, studied in 2007 and 2008; *p* < 0.001). However, the general stands consecution of different forest zones remains unchanged in all years for both tree species (Figures 4A,B) in spite of the annual variability of these root traits in some repeatedly studied sites.

### Species-specific distribution of RTD and root tip mass in forest zones

**Figure 7:**
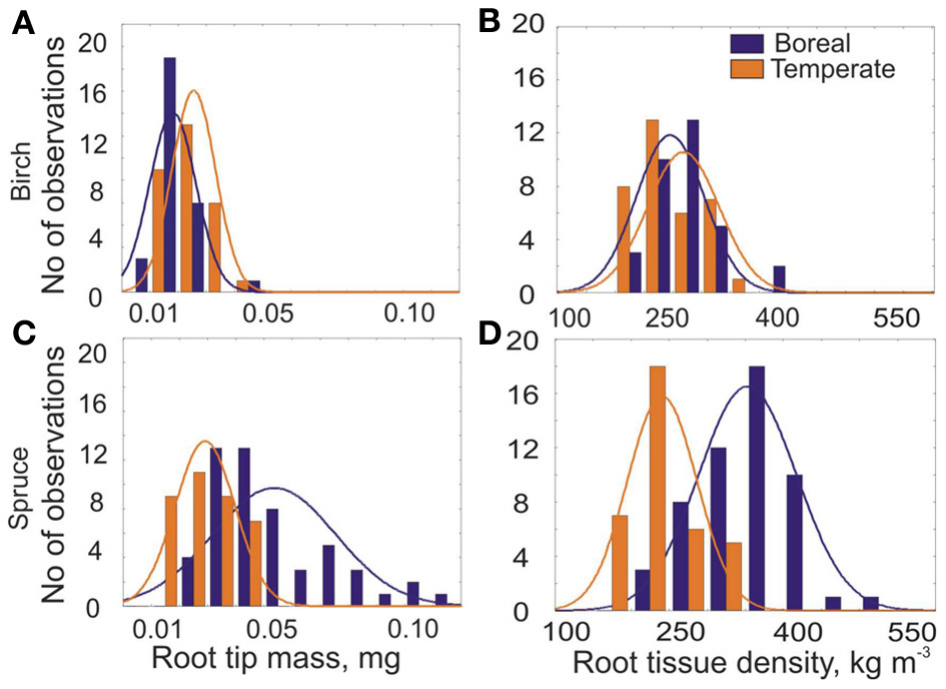
**Frequency distribution of (A and C) the mean root tip mass (W, mg) and (B and D) root tissue density of EcM roots (RTD, kg m^−3^) for two northern birch stands in Siberia (Syktykvar55, Syktykvar30) and for two southern birch stands in UK and Estonia (Risley Moss, Erastvere45) and for two most northern (Kivalo and Pallasjärvi in Finland) and two most southern (Höglwald and Altötting in Germany) spruce sites collected in 2008 (Ostonen et al., 2011)**.

The distributions of root tip mass and root tissue density of EcM roots differed between northern boreal and temperate Norway spruce stands (Figures 7C,D), while we could not find any difference for birch EcM roots tissue density (Figure 7A). EcM roots of spruce gain higher mass and RTD in northern boreal forests, which coincides with relatively longer roots in north (Figure 4A). Although the EcM root tips of birch were also longer in the north, the root tip mass was significantly higher in the southern stands (Figure 7B). The birch root tips were heavier in south due to 1.4 times thicker root tips, while the diameter of spruce EcM roots did not differ between southern and northern stands (Figure 6A).

## Discussion

Our results strongly imply that tree species may have different acclimation-indicative root traits in response to changing environments. Birch ensures the morphological acclimation across forest zones by changing SRL of the EcM roots, which is mainly (61%) determined by the variation of diameter. The acclimation of spruce EcM roots is based mainly on the variation of root tip frequency per mass unit, which, in turn is essentially (41%) determined by the variation of root tip length. Silver birch as a pioneer species must have a broader tolerance to environmental conditions across multiple environments; however, the mean PI of all MTs did not differ between early-successional birch and late-successional spruce. Duan et al. (2013) also found that deciduous *Populus yunnanensis* and evergreen *Abies faxoniana* differed in PI-s of their key traits but not in their overall plasticity. The difference between birch and spruce in the plasticity response of EcM roots and in their most indicative root trait across climate gradient shows the existence of equivalent morphological acclimation strategies in terms of optimal foraging. The clearly higher similarity in root traits of birch and spruce in temperate forest compared to boreal forests can be explained by more fertile and closer to optimal growing conditions.

Although, we considered the MTs of EcM short root tips in general, without specifying the morphotype, the species of fungal symbionts may have significant impact to shape of root tips (Ostonen et al., 2009; Makita et al., 2012). In our previous study, we have reported a shift in dominating colonizers of root tips in spruce forests across a latitudinal gradient (Ostonen et al., 2011), this might be one of the reasons behind changes in EcM root traits, such as root tip frequency in spruce or SRL in birch. However, the increase in root tip frequency may occur also due to differences in host tree genotype in northern compared to southern forests, as Korkama et al. (2006) have shown higher root tip frequency per fine root biomass for fast-growing spruce clones, which might be the case of southern Norway spruce forests. Furthermore, the increase in fine root RTF of fast growing clones coincided with changes in EcM community structure (Korkama et al., 2006). Thus, the shifts in EcM communities may play an important role in morphological acclimation and mineral nutrition of trees and should be studied in the future. Nevertheless, the alterations in EcM root morphology is one of the important mechanisms in forest trees root system acclimation to changing environments and the elucidation of general patterns of different tree species is important for predicting the ability of different species to acclimate to global change.

### Changes in EcM morphology across forest succession stages reflect root-rhizosphere level succession

The biggest part of the morphological variation of birch EcM roots was described by SRL, which has been used as an indicator of environmental change, characterizing also the economic aspects of the root system and being sensitive to nutrient availability of trees (Ostonen et al., 2007a,b; Rosenvald et al., 2011b). Morphological plasticity includes the possibility to induce changes in physiology or activity (e.g., the metabolism) of root tips (Useche and Shipley, 2010; Makita et al., 2011). Also our findings about birch indicate that change in SRL of EcM roots gives probably the biggest physiological effect in water and nutrient uptake, because it's the highest phenotypical plasticity among studied traits. SRL was high in conditions of resource deficiency, when trees have limited amount of assimilates to invest in EcM roots, but they need to increase the absorbing surface—that is to grow thin and long roots—to acquire sufficient amounts of nutrients and water. In line with our hypothesis, achieving high SRL of EcM roots is an acclimation strategy of silver birch in stress and unfavorable growing conditions (e.g., in subarctic forests) and at younger age. Our results are in good accordance by Kalliokoski et al. (2010), who showed the increase of SRL from the most fertile sites to the least fertile sites even for fine roots (<2 mm) of *Betula pendula*.

Plasticity indices of the functional parameters (SRA, SRL, RTD) were considerably higher also in the younger age group (≤10 years) compared to older silver birches. High plasticity and high SRL as well as SRA at younger age are essential, because trees have to acclimate and grow quickly to survive in competition for light, nutrients, and other resources.

Plasticity indices of root traits of stands belonging to the naturally regenerated fertile silver birch native forest chronosequence reflect mainly age-driven plasticity, because growing conditions remain relatively stable along the chronosequence (Rosenvald et al., 2013). In the case of primary succession as occurs in the chronosequence of mine site forests, soil conditions improve tremendously in time, and plasticity of root parameters is the sum of the age-related and site-conditions-related plasticity. However, mean SRL values of EcM roots of silver birch in native forest and in mine forest are more similar in older stands. As EcM root SRL of silver birch is related to soil fertility (Rosenvald et al., 2011b), more similar soil conditions in older stands determine also more similar SRL values there. Majority of the studied native birch forests still form a separate group from mine site forests in the ordination plot based on all EcM root traits probably because of the differences in soil, as the soil formation in mining areas has just started. Furthermore, the difference between EcM root diameters in mine and native forest stands increases with age. Our results indicate that for sufficient mineral nutrition of birch the optimal SRL values are achieved by changing EcM root diameter and to lesser extent also the root tissue density. Our RDA analysis showed increase in the mean stand age across forest succession gradient toward fertile native forest sites. Considering forest successional stages across mine, field and native birch forests, the stands on afforested agricultural land locate between mine and native forests. The root morphology in mine and field forests become more similar with native forests over time.

Thus, we can say that changes in EcM root morphology reflect the root-rhizosphere level succession as the root traits seems to be tightly associated with development of soil and the rhizosphere in a wider sense.

### Can root morphology be used as an indicator of EcM root lifespan?

Root traits, such as root tissue density, diameter and SRL of EcM root tips are hypothesized to be correlated with root tips lifespan (Withington et al., 2006). The differences in the distribution of mean mass and tissue density of EcM roots show distinct EcM root populations in north and south for spruce, but not for birch, though there was tendency for higher average RTD of EcM roots in northern birch stands. Higher amount of EcM roots with bigger root tissue density might indicate longer lifespan of EcM roots in northern forests. The median longevity for roots in the <0.5 mm diameter group, consisting in majority of EcM roots (Withington et al., 2006), has been reported to be more than two times longer in southern Sweden (Hansson et al., in press) compared to south-east Germany (Gaul et al., 2009). Our morphological analysis of EcM roots shows that spruce trees in northern boreal forests increase the persistence of existing EcM roots instead of forming new root tips, which also leads to the increase in root tip mass and tissue density. This might be related to fine root foraging strategies of trees, which is expressed through higher biomass of EcM roots in nutrient-poor soils of northern boreal spruce forests (Helmisaari et al., 2009; Ostonen et al., 2011) and lower turnover rate of fine roots as reported at higher latitudes (Yuan and Chen, 2010; Finér et al., 2011). In grasses, the high root tissue density has been associated with stressed environments (Craine et al., 2001). Nevertheless, the absence of differences in EcM root tissue density distributions between northern and southern birch forests and even higher average EcM root tip mass in southern stands might show either the species-specific differences in root morphology between the pioneer- and late-successional tree species and/or no difference in longevity of EcM root tips across the latitudinal gradient of the birch stands.

In conclusion, we can say that morphological parameters of EcM roots in Norway spruce and silver and downy birch, particularly the SRL, root tip frequency per mass unit as well as the mean diameter and length of the root tips, become more similar toward southern forests, which can be considered as more fertile and closer to optimal growing conditions compared to boreal forests. Furthermore, the alteration of EcM roots morphology is part of the acclimation process of birch trees, which spread in the first stage of primary and secondary successional forest areas, reflecting probably the simultaneous succession in soil, at root-rhizosphere level.

### Conflict of interest statement

The authors declare that the research was conducted in the absence of any commercial or financial relationships that could be construed as a potential conflict of interest.
