# Supplementary material for: Morphological plasticity of ectomycorrhizal short roots in Betula sp and Picea abies forests across climate and forest succession gradients: its role in changing environments
Source: Front Plant Sci. 2013 Sep 2;4:335. doi: 10.3389/fpls.2013.00335 (PMC3759007; doi:10.3389/fpls.2013.00335)
Supplement: Supplementary file 1 [file DataSheet1.DOC]

Table 1. Location, regeneration or plantation year of stand, study year, abbreviation of stand, and studied species of birch stands. * - stands included in chronosequenses used in the analyses of age-driven morphological plasticity. The superscripts note published data – ^1^ – Rosenvald et al. 2011a, ^2^ – Rosenvald et al. 2011b, ^3^ – Rosenvald et al. 2013, ^4^ – Parts et al., submitted.

| **Land type according to previous land use** | **Biotic region** | **Latitude** | **Longitude** | **Site** | **Regeneration/ plantation year** | **Study year** | **Abbreviation (letters of site name and stand age)** | **Species** |
| --- | --- | --- | --- | --- | --- | --- | --- | --- |
| Native forest land | Boreal | 66,20 | 26,40 | Kivalo | 1930 | 2006, 2009 | Ki76, Ki79 | *Betula pendula* |
|  |  | 62,18 | 50,55 | Sõktõkvar | 1994 | 2009 | SyIII-15 | *Betula pendula* |
|  |  | 62,18 | 50,55 | Sõktõkvar | 1979 | 2009 | SyII-30 | *Betula pendula* |
|  |  | 62,15 | 50,39 | Sõktõkvar | 1954 | 2009 | SyI-55 | *Betula pendula* |
|  |  | 61,49 | 29,19 | Punkaharju | 1976 | 2009 | Pu33 | *Betula pubescens* |
|  |  | 61,14 | 21,28 | Olkiluoto | 2000 | 2009 | Ol9 | *Betula pendula* |
|  | Hemi-boreal | 58,42 | 26,45 | Voore | 1939 | 2006 | Vo67 | *Betula pendula* |
|  |  | 58,37 | 27,02 | Alatskivi I * | 1977 | 2008, 2009^3^ | AkI-31, AkI-32 | *Betula pendula* |
|  |  | 58,33 | 27,05 | Alatskivi II * | 1995 | 2008, 2009^3^ | AkII-13, AkII-14 | *Betula pendula* |
|  |  | 58,19 | 21,59 | Kuusnõmme | 1914 | 2006 | Ku92 | *Betula pendula* |
|  |  | 58,15 | 27,17 | Järvselja * | 2006 | 2009^3^ | Jä3 | *Betula pendula* |
|  |  | 58,15 | 27,17 | Järvselja * | 2003 | 2009^3^ | Jä6 | *Betula pendula* |
|  |  | 58,02 | 26,01 | Aakre * | 1948 | 2008^3^ | Aa60 | *Betula pendula* |
|  |  | 57,58 | 26,56 | Erastvere * | 1964 | 2008, 2009^3^ | Er44, Er45 | *Betula pendula* |
|  | Temperate | 53,25 | 2,30 | Risley Moss | 1974 | 2009 | RM35 | *Betula pendula* |
| Oil shale mining refuse | Hemi-boreal | 59,23 | 27,13 | Kohtla-Järve | 2002 | 2005, 2006 | Kj5, Kj6 | *Betula pendula* |
|  |  | 59,19 | 27,04 | Aidu * | 1967 | 2006^2^, 2007 | Ai40, Ai41 | *Betula pendula* |
|  |  | 59,18 | 27,46 | Narva I * | 2002 | 2007^2^ | NaI-7 | *Betula pendula* |
|  |  | 59,15 | 27,42 | Narva II * | 2005 | 2005^2^, 2006^2^, 2007 | NaII-2, NaII-3, NaII-4 | *Betula pendula* |
|  |  | 59,15 | 27,42 | SirgalaII * | 1987 | 2006 | SiII-20 | *Betula pendula* |
|  |  | 59,15 | 27,42 | SirgalaI * | 1978 | 2006^2^, 2007 | SiI-29, SiI-30 | *Betula pendula* |
| Agricultral land | Hemi-boreal | 58,98 | 22,55 | Reigi | 1998 | 2006^1^, 2007 | Re8, Re9 | *Betula pendula* |
|  |  | 58,93 | 24,53 | Rapla | 1992 | 2006^1^, 2007 | Ra14, Ra15 | *Betula pendula* |
|  |  | 58,28 | 26,97 | Haaslava | 1994 | 2006^1^ | Ha12 | *Betula pendula* |
|  |  | 58,24 | 27,29 | Rõka | 2007 | 2007^4^, 2009^4^, 2010^4^, 2011^4^ | Rõ2, Rõk4, Rõ5, Rõ6 | *Betula pendula* |
|  |  | 58,23 | 25,63 | Kassi | 1995 | 2006^1^ | Ks11 | *Betula pendula* |
|  |  | 58,14 | 26,44 | Kambja | 1996 | 2006^1^, 2007, 2008 | Ka10, Ka11, Ka12 | *Betula pendula* |
|  |  | 58,03 | 27,15 | Lutsu | 1992 | 2006^1^ | Lu14 | *Betula pendula* |
|  |  | 57,88 | 26,27 | Väljaküla | 1993 | 2006^1^ | Vä13 | *Betula pendula* |
|  |  | 53,14 | 4,01 | Bangor | 2004 | 2009 | Ba7 | *Betula pendula* |

Table 2. Location, biotic region, study year and soil C/N ratio of Norway spruce stands (in age from 30 to 140 years). The superscripts note published data - ^1^Ostonen et al 2011, ^2^ Leppälammi-Kujansuu et al 2013.

| **Biotic region** | **latitude** | **longitude** | **Site** | **Study year** | **Abbreviation (3 first letters of site name and stand age)** | **Soil C/N** |
| --- | --- | --- | --- | --- | --- | --- |
| Boreal | 67,6 | 24,14 | Pallasjärvi | 2007, 2008^1^ | Pal07, Pal08 | 47 |
| Boreal | 66,2 | 26,38 | Kivalo | 2007, 2008^1^ | Ki07, Ki08 | 44 |
| Boreal | 64,07 | 19,27 | Flakaliden | 2009^2^ | Fla09 | 33 |
| Boreal | 63,33 | 22,29 | Uusikaarlepyy | 2007, 2008^1^ | Ukl07, Ukl08 | 27 |
| Boreal | 61,52 | 24,18 | Juupajoki | 2007, 2008^1^ | Ju07, Ju08 | 28 |
| Boreal | 60,38 | 23,48 | Tammela | 2007, 2008^1^ | Ta07, Ta08 | 31 |
| Hemi-boreal | 58,42 | 26,45 | Voore | 1994, 2005, 2007, 2008^1^ | Vo94, Vo05, Vo07, Vo08 | 23 |
| Hemi-boreal | 58,19 | 21,59 | Kuusnõmme | 2005, 2007 | Ku05, Ku07 | 15 |
| Hemi-boreal | 58,15 | 27,15 | Järvselja | 2008^1^ | Js08 | 27 |
| Temperate | 50,08 | 11,52 | Waldstein | 2008^1^ | WS08 | 23 |
| Temperate | 49,58 | 11,48 | Goldkronach | 2008^1^ | GLO08 | 22 |
| Temperate | 49,45 | 12,23 | Flossenbürg | 2008^1^ | FLO08 | 25 |
| Temperate | 48,17 | 11,04 | Höglwald | 2008^1^ | HOG08 | 23 |
| Temperate | 48,13 | 12,44 | Altötting | 2008^1^ | ALT08 | 27 |
